# Moderating Effects of the Ego-Energy in Relation to Stress, Drinking Motives, and Depression in Korean Adult Males

**DOI:** 10.3389/fpsyg.2021.636318

**Published:** 2021-05-20

**Authors:** Doohah Yoon, Hyonggin An

**Affiliations:** ^1^BK21PLUS Health Sciences, Korea University, Seoul, South Korea; ^2^Department of Biostatistics, College of Medicine, Korea University, Seoul, South Korea; ^3^Department of Health Management and Policy, College of Public Health, Korea University, Seoul, South Korea

**Keywords:** stress, drinking motives, depression, ego-energy, ego-gram, structural equation model

## Abstract

Although there have been numerous studies using stress and coping theories to explain the relationship between stress, drinking motives, and depression, few of them have attempted to verify these theories against adult male data. There is also a shortage of Korean studies, both theoretical and empirical, on the role of ego-energy as a moderating variable in the relationship between stress, drinking motives, and depression. This study uses a multiple-group analysis to investigate the moderating effects of the ego-energy on the aforementioned relationship in adult males. A transactional analysis tool showing the total amount of ego-energy called Ego-gram is used in this study. The tool reflects personality traits based on ego-energy state structural and functional analyses. The researchers collected empirical data to test the research model by conducting an online survey of adult males aged 20–50, residing in seven metropolitan cities and in the Gyeonggi Province. The survey yielded 567 samples. The data were then analyzed through structural equation modeling to understand the relationship between various factors. The results showed that, first, stress was positively correlated with drinking motives. Second, drinking motives had some influence on depression. Third, stress, drinking motives, and depression had statistically significant relationships between low-ego-energy and high-ego-energy groups. Overall, this study found a difference between the two ego-energy groups concerning the relationship between stress, drinking motives, and depression. Based on these results, practical implications were discussed as to how to strengthen the ego-energy, while also presenting future research directions to shed light on the precise mechanism of depression. This study is significant for exploring ways in which adult males cope with and prevent stress and depression, while also offering the basic data for improving the mental and physical health of adult males. This study is also significant for drawing attention to the necessity of developing various health and wellness programs adapted to the needs of this specific population segment while providing the data that may serve as the basis for developing such programs.

## Introduction

In this present rapidly evolving society, people face an unprecedented variety of health issues, and along with the rise in health problems came a heightened interest in health. Moreover, there has been a comparable progress in the field of public health and social science relevant to the ability of our society to meet the health care and wellness needs of its population. Citing a special report on depressive disorders prepared by Secunda et al. (1973) for the US National Institute of Mental Health, Beck et al. (1979) stated that 75% of all mental health cases treated in an inpatient care setting were related to depression, while severe depression affected 15% of all adults aged 18–74 annually. Depression would go on to cost the American society an estimated rate of US$ 300–900 million per year. In 2015, the Korea Neuropsychiatric Association conducted a mental health and happiness survey of 1,000 men and women, aged 20–59, residing in Seoul and in six metropolitan cities (i.e., Busan, Incheon, Daegu, Daejon, Gwangju, and Ulsan). Among the survey respondents, 33% answered that they had experienced emotional stress, while 56% responded that they had suspected themselves of having depression (Lee, 2015). A salient characteristic of stress research is the heavy focus on environmental changes—in other words, daily life events. Stress research deals with daily life events that influence specific health outcomes such as schizophrenia, depression, cancer, elderly mortality, and cardiovascular diseases (Lazarus and Folkman, 1984). Lazarus and Folkman (1984) described coping with stressful situations as constantly changing cognitive and behavioral efforts to manage specific external or internal demands that are appraised as taxing or exceeding the resources of the person (Oh and Shin, 2016).

In general, Ego-gram helps to relate the stress and coping mechanisms of an individual to total ego-energy. Specifically, the Ego-gram and its underlying theory were developed in the mid-1950s by the United States psychiatrist Berne (1957) based on a psychoanalytic and group therapy research. The purpose of Ego-gram is to classify individuals according to their personality traits. The Ego-gram is a transactional analysis (TA) psychotherapy tool that helps evaluate and decide the usefulness of personal growth and health through changes in the ego-energy states, which are the critical components of the personality structure of an individual. The personality structures correspond to various ego-energy states postulated in the transactional theory. As a self-analysis tool based on the transactional theory, the Ego-gram expresses the dynamics between various parts of a personality structure and the amount of psychic ego-energy that is released into the exterior. These values are expressed in terms of three ego-energy states (P, A, and C) and five functional ego-energy states (CP, NP, FC, AC, and A), in the form of a bar graph.

All human beings contain three ego-energy types within themselves. An ego-energy state is defined as a system of thoughts and feelings with a set of related behavior patterns (Dusay, 1977). One of the three ego-energy states is the parent ego-energy state (P)—divided into critical parent (CP) and nursing parent (NP) ego-energy states—which is absorbed or copied from the parents. The second is the adult ego-energy state (A), the state in which individuals respond to reality as an adult through thought and analysis. The third one is the child ego-energy state (C), the state of the ego-energy in which individuals respond to reality like a child according to their feelings. This ego-energy is further divided into free child (FC) and adapted child (AC) ego-energy states (Kim, 1994). The higher the score in the test, the larger the total amount of ego-energy, while the lower the score, the smaller the amount of ego-energy. The study by Dusay (1977) determined the relationship between the different ego-energy states and the amount of psychic energy that is released externally to understand how the personality of an individual manifests itself through a particular behavior pattern (Kim, 2013). Not only does the Ego-gram reflect the various characteristics and tendencies of an individual, but it can also be used to develop educational programs to change behaviors (An and Lee, 2007; Barnes, 2007). Ego-gram results are widely used in fields such as education, psychology, and psychiatry as essential reference data because they provide suggestions for qualitative improvement (Kim, 2009). The Tokyo University Ego-gram developed by the Tokyo University is notable for analyzing the ego-energy state structure to help them understand the influence of personality traits on the health conditions of diabetic and high-blood-pressure patients and general working adults (Nakkao et al., 1999; Shimizu and Fujii, 2001). However, any attempt to empirically analyze the relationship between stress, drinking motives, and depression in adult males aged 20–50, using the ego-energy as a moderating variable, is scarce.

At the same time, the relevance of this study was supported by the studies such as the reports by Lee (1997) on the relationship between the ego-energy, self-esteem, and other variables; Park and Rhee (2005) on stress, drinking, and drinking motives; and Kim (2017) on the relationship between stress, depression, and drinking motives.

There have been studies investigating the relationship between some of the factors related to drinking, such as stress, drinking motives, self-esteem, ego-energy, or ego-energy resilience. Also, several comprehensive studies have been conducted on the relationship between drinking motives and depression. Earlier studies dealing with topics similar to this study, such as Kim and Kim (2014), have established the influence of ego-energy resilience on the vulnerability of individuals to depression. Accordingly, in this study, the researchers set up the hypothesis that the amount of ego-energy acts as a moderating variable in the relationship between stress, drinking motives, and depression in adult males.

These studies showed that the ego affects stress, drinking, and depression. It would be important to understand the moderation effects between stress and drinking and between drinking and depression at the same time, so that we may come up with different approaches for the different ego-energy statuses to deal with drinking and depression problems if moderation effects exist. Adult males in South Korea have been under high-stress professional and comparative environments and have heavy drinking habits. Besides, it is also interesting to examine the moderating effect of ego-energy on Korean males who are different from males in other countries since ego-energy reflects cultural tradition and social norms such as conservativeness and modesty standards.

Thus, it is important to investigate the role and significance of the “total amount of ego-energy” variable to clearly understand the nature and intensity of the relationship between stress, drinking motives, and depression in Korean adult males.

The goal of this study is to determine the total amount of ego-energy in Korean adult males to provide primary data for creating an effective predictive model. It is expected that the results of this study will not only provide additional criteria for the diagnosis of depression but also serve as basic strategic data for preventive efforts. The ultimate significance of this study suggests practical strategies for improving the mental health and psychological well-being of Korean adult males.

Based on the theory by Cohen et al. (1983), as well as by Lazarus and Folkman (1984), the current study aims to investigate how stress affects drinking motives in Korean adult males and the moderation effect of ego-energy on drinking motives that influence depression. Testing the moderation effect of ego-energy on the relationship between stress, drinking motives, and depression will help reduce the influence between stress, drinking motives, and depression through various interventions following their levels.

## Materials and Methods

### Participants

This research analyzed the data from “Research on Stress, Drinking Motive, Depression, and Ego-energy among Korean Male Adult Population” collected for a month between May and June in 2019. Data collection and arbitration were carried out after receiving approval from the Korea University Institutional Review Board (IRB) on May 15, 2019 (Approval Number: KUIRB 2019-0113-02).

Data collection was conducted by the Embrain Company in South Korea, a large survey company with more than 1.3 million nationally representative panels. Samples were obtained by dividing the areas into large, medium, and small cities and then into towns and townships. Research subjects were adult males aged between 20 and 50 years dwelling in Gangnam and Gangbuk areas in Seoul, six other metropolitan cities (i.e., Busan, Daegu, Daejeon, Gwangju, Incheon, and Ulsan), and Gyeonggi-do. Sampling was carried out by equally distributed age groups. In these strata, samples were selected by random sampling. The company sent email invitations to selected panels. If panels met the eligibility criteria and consented to participate in the survey, they were asked to fill out the questionnaires by email. The company maintained the data integrity. The panels were given a 112-item survey, with 74 items covering stress, drinking motives, depression, and ego-energy. The survey was conducted from June 17 to June 19 in 2019. Among 625 responses based on the previous survey results, 567 responses were collected except for incomplete responses.

### Model and Hypothesis Building

**FIGURE 1:**
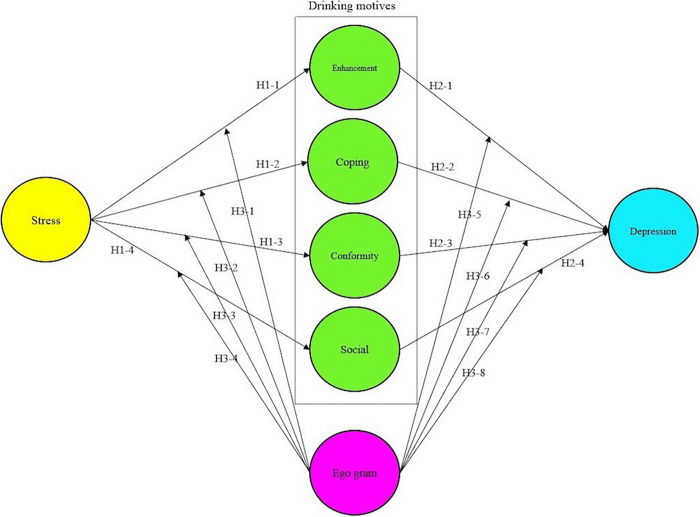
Diagram of the research model.

This study analyzes the influence between stress, drinking motives, and depression and the moderation effect of ego-energy among adult males aged between 20 and 50 by theoretically derived relating variables. While stress is an exogenous variable, drinking motives and depression are endogenous variables. The mental and psychological latent variable of ego-energy is a moderating variable. The following conceptual model (Figure 1) was created based on a review of related studies.

This study determines the effects of stress (allegedly a major trigger of depression), drinking motives, and depression on adult males. It also poses hypotheses and proposes another path, such as the moderation effect of ego-energy, which will have a significant and strong influence compared to analyzing the relationships between stress, drinking motives, and depression alone. It is expected that the moderation effect of the ego-energy will be made clear based on the previous studies. Causal relations and directionality between latent factors suggested in a study model were examined using a one-tailed test between relationship hypotheses. The hypotheses of this study are as follows:

Hypothesis H1. Stress in adult males is positively correlated with drinking motives (+).

H1-1. Stress in adult males is positively correlated with enhancement motives (+).
H1-2. Stress in adult males is positively correlated with coping motives (+).
H1-3. Stress in adult males is positively correlated with conformity motives (+).
H1-4. Stress in adult males is positively correlated with social motives (+).

Hypothesis H2. Drinking motives in adult males are positively correlated with depression (+).

H2-1. Enhancement motives in adult males are positively correlated with depression (+).
H2-2. Coping motives in adult males are positively correlated with depression (+).
H2-3. Conformity motives in adult males are positively correlated with depression (+).
H2-4. Social motives in adult males are positively correlated with depression (+).

Hypothesis H3. Stress, drinking motives, and depression in adult males have a difference among ego-energy groups.

H3-1. Stress and enhancement motives in adult males have a difference among ego-energy groups.
H3-2. Stress and coping motives in adult males have a difference among ego-energy groups.
H3-3. Stress and conformity motives in adult males have a difference among ego-energy groups.
H3-4. Stress and social motives in adult males have a difference among ego-energy groups.
H3-5. Enhancement motives and depression in adult males have a difference among ego-energy groups.
H3-6. Coping motives and depression in adult males have a difference among ego-energy groups.
H3-7. Conformity motives and depression in adult males have a difference among ego-energy groups.
H3-8. Social motives and depression in adult males have a difference among ego-energy groups.

### Measuring Instruments

For this present research method, an online survey was conducted through an institution specialized in academic research. The measuring instruments (survey questions) adopted in this study were based on prior research reliability and validity and were modified and verified for this research. These measuring instruments reveal influencing factors between stress, drinking motives, and depression among adult males aged 20s–50s. This quantitative research study is determined by comparing and analyzing whether there is a statistically significant or strong influence attributed to significant ego-energy differences. Tables 1, 2 provide the number of items and possible range of scores and correlations among study variables used in the analysis.

**TABLE 1:** Number of items and range of scores for variables.

**Variable**		**Number of items**	**Range of scores**
Stress		5	5 Likert’s scale/5–25
Drinking motives	Enhancement motives	4	5 Likert’s scale/16–20
	Coping motives	4	
	Conformity motives	4	
	Social motives	4	
Depression		3	4 Likert’s scale/3–12
Ego		50	5 Likert’s scale/50–250

**TABLE 2:** Correlations among study variables (*N* = 567).

	**Stress**	**Enhancement motives**	**Coping motives**	**Conformity motives**	**Social motives**	**Depression**
Stress	1					
Enhancement motives	0.33	1				
Coping motives	0.46	0.56	1			
Conformity motives	0.39	0.60	0.40	1		
Social motives	0.14	0.63	0.35	0.52	1	
Depression	0.65	0.25	0.32	0.27	0.07	1

#### Stress

Regarding a stress-related scale in this study, Yim et al. (1996) revised the Brief Encounter Psychosocial Index (BEPSI) developed by Frank and Zyznaski (1988). The BEPSI-Korean version (BEPSI-K) completed with validity was used by Cha et al. (2017) and was therefore used in this study as well. The instrument consists of five items, with each item using the Likert scale from 1 to 5. The measurement tool was designed to evaluate emotions experienced during the previous month on a scale of 1–5, with 1 meaning “never” and 5 meaning “always.” Example questions are: “Have you ever felt problems difficult to endure mentally or physically?” and “Have you ever forgotten important tasks due to so many things to do?” Stress levels experienced during the previous month are estimated using the possible total score ranges from 5 to 25, with a higher score signifying higher stress levels. The reliability of the study by Cha et al. (2017) (Cronbach’s α) was 0.87 for men and 0.88 for women. The internal consistency of this study is 0.86 for men only.

#### Drinking Motives

Cox and Koinger (1990) devised a scale to estimate various drinking motives. This study uses a scale that follows the study by Cox and Koinger (1990), as well as the scales used by Shin (1998) and Shin and Han (1999). The scale for drinking motives was designed to estimate four factors, including enhancement, coping, conformity, and social motives from two dimensions, such as directionality and a source. Each drinking motive was composed of 16 items, with four items per four factors. Each question, which can be answered using a five-point scale ranging from “never drink” to “always drink,” determines the reason for drinking and assesses drinking frequency under specific circumstances indicated by each question. Higher scores mean a higher probability to drink alcohol. The motive generated to produce positive emotions and well-being is an enhancement motive. Coping motives are produced to cut down negative emotions or adjust them. Conformity motives are generated to avoid social criticism or rejection. Social motives are produced to gain positive social guarantees. In terms of reliability (Cronbach’s α), the study by Shin and Han (1999) showed that enhancement motives were 0.76, coping motives were 0.89, conformity motives were 0.80, and social motives were 0.85. For the reliability of this study (Cronbach’s α), four questions regarding enhancement motives are 0.76, four questions regarding coping motives are 0.90, four questions regarding conformity motives are 0.80, and finally, four questions regarding social motives are 0.84.

#### Depression

This study builds on previous studies by revising their scales to measure depression levels. The National Institute of Mental Health (NIMH) developed the Center for Epidemiologic Studies Depression (CES-D) scale in 1971 to measure depression levels. Radloff (1977) developed a scale based on the CES-D, which was then used by Weissman et al. (l986), Cho and Kim (1993), and Lee and Kahng (2009). Depression levels were also measured using self-reported depression scales consisting of 20 questions, a primary instrument used by Radloff (1977) and Weissman et al. (l986) for diagnosing depression. This study also adopts depression scales analyzed and used by Lee and Kahng (2009) and CES-D scales which measured depression levels using 11 questions that used the Likert scale ranging from 1 to 4. Using the same 11 questions with the Likert scale, the study by Cho and Kim (1993) had a reliability (Cronbach’s α) of 0.91, Kim et al. (2015) had a reliability (Cronbach’s α) of 0.88, Lee and Kahng (2009) had a reliability (Cronbach’s α) of 0.87, and Kim and Gil (2016) had a reliability (Cronbach’s α) of 0.91. In this study, we used CES-D 11 items and had a reliability (Cronbach’s α) of 0.87.

#### Ego-Energy (Personality Structure, Ego-Energy Amounts, Ego-Energy Status)

The ego-energy (TA) is a personality theory developed by an American psychiatrist Berne (1910–1970) in the 1950s. The Korea Transactional Analysis Association (KTAA) standardized it as a personality questionnaire in 1996. The Ego-gram is an indicator of the total energy amount of five ego-energies in psychoanalysis. The Ego-gram developed by Dusay (1977) was adopted with the use of the Ego-gram checklist standardized by the KTAA to measure the ego-energy status. This Ego-gram classifies personality traits according to five types: critical parent (CP), nurturing parent (NP), free children (FC), adapted child (AC), and adult (A). CP represents criticizing, controlling, and dominant tendencies. NP is related to assistance, care, and encouragement. An A means that the adult ego-energy can judge in a real and rational way. FC represents the ego-energy status of a child with creativity and freedom. AC is defined as the ego-energy of a child under oppression and adaptation. The scale is composed of 50 questions by 10 ones per five functions. Each question uses a 5-point scale ranging from “always” to “never.” The score ranges from 0 to 50, with higher scores signifying higher ego-energy.

In terms of measuring the ego-energy, the scale reliability (Cronbach’s α) of the study by Woo (1997) amounted to 0.74, and its content validity was 0.84. According to the studies by Lee (1997) the reliability (Cronbach’s α) was 0.71, Kim (2013) 0.84, Won and Kim (2002) 0.8379, Lee (2006) 0.87, and Hur and Cho (2014) it was 0.74, with a content validity of 0.84. The reliability of this study (Cronbach’s α) corresponded to 0.853. After verifying reliability and validity based on subfactors presented by previous researchers, they are aggregated with validity analysis results showing the reliability (Cronbach’s α) of 0.85. The researchers concluded that 50 questions were reduced to two moderating variables *via* analysis.

#### General Traits of Adult Males

The demographic variables include age, geographical residence, occupation, religion, education levels, yearly household income, marital status, subjective health status, and disease outcome. The demographic age of adult males ranges from 20 to 59 years. Their geographical residence encompasses Gangnam and Gangbuk areas in Seoul, the six metropolitan cities (i.e., Busan, Incheon, Daegu, Daejon, Guangju, and Ulsan), and Gyeonggi-do. Their education levels are classified into a high-school diploma, a bachelor’s degree, or a master’s degree. The socioeconomic variables include the yearly household income. Participants can indicate their standard annual income by using an interval scale with a unit of KRW10 million before tax, including annual salary and bonuses. The distribution of respondents by yearly household income is divided into five. Marital status is classified into married and unmarried. The subjective health status is evaluated using a 5-point scale ranging from “very healthy” to “very unhealthy.” The disease outcome was evaluated according to the following items: no disease, cerebrovascular disease, cardiac disease, diabetes, arteriosclerosis, high blood pressure, and other diseases. Table 3 shows the general traits of the participants.

**TABLE 3:** Demographic distribution (*N* = 567).

**Characteristics**		**Frequency (*N* of samples)**	**Percentage (%)**
Age	20’s	142	25.0
	30’s	136	24.0
	40’s	143	25.2
	50’s	146	25.7
Geographical residence	Gangnam Area	95	16.8
	Ganbuk Area	97	17.1
	Six metropolitan cities	191	33.7
	Gyeonggi-Do	184	32.5
Occupation	Salary man	193	34.0
	Blue-collar worker	61	10.8
	White-collar worker	163	28.7
	Technical worker	60	10.6
	Business and Management	60	10.6
	Professional	30	5.3
	Protestant	100	17.6
Religion	Roman Catholic	56	9.9
	Buddhism	74	13.1
	No Religion	337	59.4
Education	High School Diploma	63	11.1
	Bachelor’s Degree	425	75.0
	Master’s Degree and Over	79	13.9
Yearly Income (KRW)	Less than 30 mm	68	12.0
	From 30 to 40 mm	129	22.8
	From 40 to 50 mm	89	15.7
	From 50 to 60 mm	112	19.8
	60 mm and over	169	29.8
Marriage	Married	339	59.8
	Unmarried	228	40.2
Health status	Very health	35	6.2
	Healthy	223	39.3
	Normal	263	46.4
	Not healthy	45	7.9
	Very unheathy	1	0.2
Disease status	No disease	387	68.3
	Cerebrovascular disease	2	0.4
	Cardiac disease	4	0.7
	Diabetes	18	3.2
	Arteriosclerosis	1	0.2
	High blood pressure	90	15.9
	Other disease	65	11.5
	Total	567	100.0

*KRW, South Korean won. mm, 1,000,000.*

#### Statistical Analysis

The sociodemographic characteristics of the respondents were determined *via* descriptive statistical analysis. A frequency analysis, reliability analysis, and Pearson item-total correlation analysis were implemented to compare and analyze the subfactors of major variables following the demographic characteristics of the samples. AMOS was used in confirmatory factor analysis (CFA) to verify the validity of the study models. A structural equation model was implemented to verify the hypothesis of the study models. The model helped to test the interrelation between constructs. A covariance structural model was used to test the relationship between stress, drinking motives, depression, and ego-energy. The path and statistical significance were reviewed at the final structural model to determine the influence of stress, drinking motives, and depression. In order to evaluate the model fit, we utilized commonly used goodness-of-fit indices such as Root Mean-square Residual (RMR, goodness of fit < 0.08), such as Goodness of Fit Index (GFI, goodness of fit > 0.85), a Comparative Fit Index (CFI, goodness of fit > 0.85), a Normed Fit Index (NFI, goodness fit > 0.85), and a Root Mean Square Error of Approximation (RMSEA, goodness of fit < 0.08). For evaluation of factor influence on variables, factor loadings were examined (good influence > |0.4|). For discriminant validation, we used Average Variance Extracted (AVE) values. We also examined a Chronbach’s α (>0.6 is acceptable) for internal consistency. For the evaluation of the moderation effect of ego-energy, we first standardized the ego-energy scores as follows: $\frac{\mathrm{Ego}-\mathrm{energy}\,\text{score}-\text{ego}-\text{energy}\,\text{mean}\,\text{score}}{\text{SD}\,\text{of}\,\mathrm{ego}-\mathrm{energy}\,\text{score}}$. Then, we categorized the ego-energy into two groups: the high-ego-energy group if the standardized score is 0 or higher and the low-ego-energy group if the standardized score is lower than 0. Regarding the relationship between stress, drinking motives, and depression, multiple group analysis, and *χ*^2^ difference tests were conducted to compare and verify the difference measurement invariance among ego-energy groups. A maximum likelihood method was used for the path coefficient. We used Bonferroni corrections to adjust the nominal level of significance due to multiple comparisons. The significance level was set at 0.05 divided by the respective number of hypothesis tests performed in each analysis. The data were analyzed using SPSS version 21.0 and AMOS 21.0 (SPSS Inc., Chicago, IL, United States).

## Results

### Testing the Reliability and Validity of the Constructs

The confirmatory factor analysis was carried out to test the validity and reliability of the constructs and the result is as follows. The fit index of measurement models results in RMR = 0.045, GFI = 0.895, CFI = 0.922, NFI = 0.892, and RMSEA = 0.063. These models were deemed satisfactory. The factor loading of all measured items was ≥0.546. The AVE values for discriminant validity ranged from 0.480 to 0.802, and they were acceptable. The result of the discriminant validity test was good. The high-construct reliability was displayed because the reliability of each construct ranged from 0.620 to 0.896 (Cronbach’s α), while construct reliability per construct was in the range of 0.783–0.924.

#### Hypothesis Testing Between Stress, Drinking Motives, and Depression

The hypothesis testing was analyzed using AMOS 21.0, and the results are shown in Table 4. The major fit indices that were satisfactory are as follows: *χ*^2^ = 1,087.248 (*df* = 242, *p* < 0.001), GFI = 0.856, CFI = 0.876, NFI = 0.847, and RMSEA = 0.079.

**TABLE 4:** Hypothesis testing.

**Path**		**Estimate**	**Standard error (SE)**	***t*-value (CR)**	***p*-value**	**Standardized estimate**	**Proven or not**
H1-1	Stress → Enhancement motives	0.326	0.044	7.351	*p* < 0.001	0.437	Proven
H1-2	Stress → Coping motives	0.680	0.068	10.002	*p* < 0.001	0.505	Proven
H1-3	Stress → Conformity motives	0.468	0.057	8.212	*p* < 0.001	0.457	Proven
H1-4	Stress → Social motives	0.171	0.050	3.412	*p* < 0.001	0.168	Proven
H2-1	Enhancement motives → Depression	0.360	0.097	3.695	*p* < 0.001	0.273	Proven
H2-2	Coping motives → Depression	0.156	0.035	4.502	*p* < 0.001	0.214	Proven
H2-3	Conformity motives → Depression	0.213	0.049	4.381	*p* < 0.001	0.223	Proven
H2-4	Social motives → Depression	−0.267	0.065	−4.131	*P* < 0.001	−0.277	Proven

*CR, critical ratio; It is important to display the statistical significance of path coefficients. If the CR value is larger than ±1.965 or if the *p*-value is smaller than 0.05, it is statistically significant.*

The results of hypothesis testing show that stress affects the drinking motive. Therefore, H1 and H2, which indicate that drinking motives affect depression, were proven. Therefore, the results reveal that drinking motives influence depression.

Based on the above results, while stress has a direct effect on drinking motives, the influence of drinking motives on depression is affected by enhancement, coping, coherent, and social motives. Since depression is affected by these motives, drinking motives must be taken seriously.

#### Moderation Effect Testing of Multiple Group Ego-Energy Between Stress, Drinking Motives, and Depression

The multiple group analysis between the low-ego-energy group and the high-ego-energy group was verified to test the hypothesis that the relationship between stress, drinking motives, and depression will have a difference among more than two ego-energy groups. Table 5 shows the results, where stress is an exogenous variable, drinking motives and depression are endogenous variables, and ego-energy is a moderating variable.

**TABLE 5:** Comparison of the path coefficient among ego-energy groups of testing hypothesis on the moderation effect by ego-energy (*N* = 567).

**Path**		**Whole model**		**Group classification**				**Moderating effect**	
**Hypothe-sis Code**	**Hypothesis**	***N* = 567**		**Low ego-energy 1 group (*N* = 249)**		**High Ego-energy 2 group (*N* = 318)**		**Testing**	
		**Standardized estimate**	**Proven or not**	**Standardized estimate**	**Proven or not**	**Standardized estimate**	**Proven or not**	***t*-value (C⋅R)**	**Proven or not**
H1-1	Stress → Enhancement motives	0.437	Proven	0.560***	Proven	0.258***	Proven	1.998	Proven
H1-2	Stress → Coping motives	0.505	Proven	0.516***	Proven	0.397***	Proven	0.262	Rejected
H1-3	Stress → Conformity motives	0.457	Proven	0.474***	Proven	0.325***	Proven	–0.204	Rejected
H1-4	Stress → Social motives	0.168	Proven	0.100(0.196)	Rejected	0.130*	Proven	–0.952	Rejected
H2-1	Enhancement motives → Depression	0.273	Proven	1.983***	Proven	0.104(0.363)	Rejected	3.994	Proven
H2-2	Coping Motives → Depression	0.214	Proven	−0.432*	Proven	0.209*	Proven	–2.785	Proven
H2-3	Conformity motives → Depression	0.223	Proven	−0.051(0.487)	Rejected	0.220***	Proven	–2.320	Proven
H2-4	Social motives → Depression	–0.277	Proven	−1.620***	Proven	−0.195*	Proven	–3.765	Proven

***p < 0.001; *p < 0.05.

*CR, critical ratio; It is important to display the statistical significance of path coefficients. If the CR value is larger than ±1.965 or if the p-value is smaller than 0.05, it is statistically significant.*

The multiple group analysis of a moderation effect was tested to determine the statistically significant difference between the sizes of the path coefficients of the two groups. It was shown that the difference in path coefficients among groups had a significant effect. It was also revealed that the multiple group results of a moderation effect between stress, drinking motives, and depression among the two groups had a statistically significant effect.

In the first step, the measurement equivalence was tested using multiple group confirmatory factor analysis, with results as shown in Table 6. There were 249 and 318 subjects in the high-ego-energy group and the low-ego-energy group, respectively.

**TABLE 6:** Comparison of measurement invariance test between the free and constrained model.

**Model**	***χ*^2^**	***df***	**GFI**	**AGFI**	**CFI**	**TLI**	**RMSEA**
Free model	1,050.00	474	0.87	0.83	0.91	0.90	0.05
Constrained model	1,071.31	49	0.87	0.84	0.91	0.90	0.05

The *χ*^2^ difference was tested between a free model among groups and a constrained model among factor loading with the use of the confirmatory factor analysis. According to the results, the *χ*^2^ difference of a free model was*χ*^2^ = 1,050.00, *df* = 474, and that of a constrained model was *χ*^2^ = 1,071.31, *df* = 492. Therefore, as the degree of freedom increased to 18 in the case of a free model and a constrained model, the difference in *χ*^2^ amounted to Δ*χ*^2^ = 21.30 (*df* = 18). No significant difference was shown between the two groups. The results revealed that the measurement equivalence was secured because the CFI for the free model was 0.91 and for its constrained model it was 0.91, while the TLI for the free model was 0.90 and for its constrained model it was 0.90. Moreover, the RMSEA of the free model was 0.046 and for its constrained model, it was 0.05, with no statistically significant difference.

We evaluated the difference in path coefficients among the groups. Table 5 shows the path coefficients among the two groups ahead of analysis. The results showed that in the low-ego-energy group, the rejected hypotheses were stress social motives and conformity motive depression. However, for the high-ego-energy group, only the enhancement motive depression hypothesis was rejected.

Therefore, the statistically significant results were displayed on the path of the two groups. In other words, the ego-energy levels determined the relationship between stress, enhancement motives, coping motives, conformity motives, social motives, and depression.

Next, Δχ^2^ was used to test the χ^2^ difference between a free model and a constrained one between each path to check again whether the significant difference of path coefficients (γ) among groups in a model existed. A moderation effect was analyzed between low-ego-energy and high-ego-energy groups to check whether a difference existed among the ego-energy-based groups. Table 5 shows the results analyzed by pairwise parameter comparison [*t*-value, and critical ratio (CR)] to figure out the moderation effect between the two groups.

The researchers determined whether ego-energy had an effect as a moderating variable in the relationship between stress, drinking motives, and depression. Based on the results, a significant difference was revealed in the relationship between enhancement motives under stress, depression under enhancement motives, depression under coping motives, depression under conformity motives, and depression under social motives between the low-ego-energy group and the high-ego-energy group. No significant difference was found in the remaining hypotheses. Therefore, a partial moderation effect based on ego-energy was found between the two groups. Table 5 shows the result of testing the moderation effect on structural equation models of multiple groups between the low-ego-energy group and the high-ego-energy group. The results revealed a significant influence based on a significant difference where a difference value path among groups is more than ±1.96.

The test results per group are as follows. The moderation effect of ego-energy is found in the influence of stress on enhancement motives [*t*-value (CR) = 1.998]. In other words, the influence (γ = 0.560, *p* < 0.001) of stress on enhancement motives of the low-stress group is more significant than the influence of stress (γ = 0.258, *p* < 0.001) on enhancement motives of the high-stress group. In the low-ego-energy group, increased stress tends to raise enhancement motives (γ = 0.560), similar to the high-ego-energy group (γ = 0.258). The study determined that the effect of enhancement motives on depression has a moderation effect on ego-energy [*t*-value (CR) = 3.994]. The influence (γ = 1.983, *p* < 0.001) of enhancement motives on depression in the low-ego-energy group is more significant than the influence (γ = 0.104, *p* = 0.363) of enhancement motives on depression in the high-ego-energy group. In the low-ego-energy group, high enhancement motives tend to raise the depression levels (γ = 1.983), similar to the high-ego-energy group (γ = 0.104). The influence of coping motives on depression has a moderation effect on ego-energy [*t*-value (CR) = −2.785]. The influence (γ = −0.432, *p* < 0.05) of coping motives on depression among the low-ego-energy group is more significant than the influence (γ = 0.209, *p* < 0.05) of coping motives on depression among the high-ego-energy group. In the low-ego-energy group, increased coping motives tend to lower the depression levels (γ = −0.432), while in the high-ego-energy group, increased coping motives tend to raise the depression levels (γ = 0.209). From these results, it can be said that the influence of conformity motives on depression has a moderation effect on ego-energy (−2.320). In other words, the influence (γ = 0.220, *p* < 0.001) of conformity motives on depression in the high-ego-energy group is more significant than the influence (γ = −0.051, *p* = 0.487) of conformity motives on depression in the low-ego-energy group. In the low-ego-energy group, increased conformity motives tend to lower the depression levels (γ = −0.051), while in the high-ego-energy group, increased conformity motives tend to raise the depression levels (γ = 0.220). Based on these results, it can be considered that the influence of social motives on depression has a moderation effect on ego-energy [*t*-value (CR) = −3.765]. In other words, the influence (γ = −1.620, *p* < 0.001) of social motives on depression in the low-ego-energy group is more significant than the influence (γ = −0.195, *p* < 0.05) of social motives on depression in the high-ego-energy group. In the low-ego-energy group, increased social motives tend to lower the depression levels (γ = −1.620), similar to the high-ego-energy group (γ = −0.195).

The results show that a significant difference in the path to stress, drinking motives, and depression existed following the standard of the ego-energy-group. In light of this, efforts to enhance the ego-energy need to be made, and suggestions for health policies and strategies should also be established to enhance ego-energy.

## Discussion

In the process to identify the causal relationship between stress, drinking motives, and depression among adult males between 20s and 50s dwelling in Seoul and other Korean metropolitan areas, this study provided the basic information that enhances health by proving the moderation effect of ego-energy. To this end, a quantitative investigation method is selected, which determines the effects of stress, drinking motives, and depression. Furthermore, the moderation effect of the ego-energy is tested with measurement invariance in the interconnected relationships between stress, drinking motives, and depression. The results and some layout suggestions of this study are summarized in the following paragraphs.

First, the results show that stress has a significant effect on drinking motives, which means that stress among adult males tends to have high levels of enhancement, coping, conformity, and social motives. This result coincides with the results of studies by Kang and Kim (2014) and Kim (2017), where drinking motives and stress are positively correlated. The result also agrees with the study by Cooper et al. (1988), where consistent drinking as a coping motive weakens the ability of an individual to manage the emotions that help control stress. Lee and Chung (2011) recognized that drinking motives significantly affect personal relationships and that alcohol was used as an escape from daily reality to relieve depression and stress. Under these circumstances, changing stress tendencies is required. The results of this study also indicate the type of stress that influences drinking motives. It is necessary to emphasize the traits of stress as follows: problems are difficult to bear mentally and physically, feelings of insufficiency to live like a decent human in certain circumstances or frustrations in striving to follow their ideal life.

Second, the results show that drinking motives have a significant effect on depression. This means that adult males who are likely to drink alcohol—to achieve positive emotions and well-being, reduce negative feelings, avoid criticism and rejection from society, or acquire positive social guarantees—tend to feel depressed. This finding agrees with the findings of these studies. Depression and coping motives show a significant positive correlation in the study by Jang (2017). In the case of coping motives, depression is a significant predictor variable in the studies by Jang (2017) and Joeng et al. (2015). Depression is the variable that significantly predicts conformity motives in the study by Joeng et al. (2015). Joeng et al. (2015) insisted that depression or the inability to build new relationships tends to give in to peer pressure in terms of drinking to avoid criticism or rejection. The study by Joeng et al. (2015) explained that drinking motives and depression are deeply connected and have positive correlations. Therefore, seeking a way to change the tendency of adult males for drinking motives to reduce depression is necessary.

Third, the results revealed that ego-energy plays a mediating/moderating role between stress, drinking motives, and depression in adult males. The multiple group results of the moderating effect between the low-ego-energy and the high-ego-energy groups show the statistically significant influence on stress, drinking motives, and depression. These results are supported by the study of Sohn et al. (2011), which indicates that self-esteem controls the relationship between drinking alcohol and depression. According to the study by Sohn et al. (2011), when looking at the moderation effect of self-esteem where drinking exerts the influence on depression, self-esteem not only independently affects depression but also shows a moderation effect between drinking alcohol and depression. Self-esteem has a direct effect on depression and serves as a moderating variable that acts as a buffer to alcohol consumption. As a result, respondents with higher self-esteem tend to feel less depressed, even though their alcohol consumption is equally high. However, the results of this study show that the adult male ego-energy has a moderation effect on the relationships between stress, drinking motives, and latent variables of depression, as well as between stress and enhancement motives, enhancement motives and depression, coping motives and depression, conformity motives and depression, and social motives and depression in terms of subvariables. Adult males with high-ego-energy levels tend to make less trouble when the possibility of drinking goes up to ensure positive feelings or well-being even though their stress levels are equivalently high. Moreover, adult males with high-ego-energy, who are more likely to drink alcohol to achieve positive feelings of well-being and acquire social guarantee, feel less depressed. Adult males with low-ego-energy tend to feel less depressed even though they are likely to drink alcohol to reduce negative feelings or avoid criticism and rejection. It can be said that the ego-energy controls the relationships between stress, drinking motive, and depression because drinking motive and depression vary depending on the ego-energy level and present circumstances. Stress, drinking motives, and depression serve as the predicting factors and are therefore significant variables. Adult males under stress have varying drinking motives and depression levels based on the differing experiences in coping with stress and differences in personality and character.

The limitations of this study and suggestions for study directions are as follows: first, the study samples are limited to an adult male population in specific metropolitan regions in South Korea, so the results of this study cannot be applied and generalized to other groups. Second, only a causal model between two variables, such as stress and drinking motives or drinking motives and depression, is applied to determine factors affecting drinking motives and depression. If other belief variables, such as M and N Ego-gram personality test for character types, were added to a study model other than stress, the researchers presume that drinking motives and depression will be explained more effectively. Third, even though we modeled the causal pathway among stress, drinking motives, and depression, it cannot be determined by the current cross-sectional study. Besides, other causal pathways are possible. To examine the causal pathway rigorously, a well-designed prospective longitudinal study is required. Fourth, research and development on stress, depression, and other related health processes are necessary because psychoanalysis and mental health programs concerning stress and depression among adult males are not yet well-established. Fifth, prediction and big data analysis of mental health among populations will give important and meaningful information. A variation in the method of study is important in deriving new information regarding mental health.

## Conclusion

This study provides a model that explains stress, drinking motives, and depression of adult males through ego-energy. The results show that, in terms of a moderation effect among groups, the ego-energy has a partially significant and strong influence on the relationship between stress, drinking motives, and depression. Highlighting the need for developing a program to enhance the total amount of ego-energy in the personality structure of adult males is important. This suggests that the amount of ego-energy is necessary to the personality structure and should be considered in the relationship between their stress, drinking motives, and depression.

In testing and examining structural relations among four variables, such as stress, drinking motives, depression, and ego-energy, variable relationships are as follows. Stress is factored into an exogenous variable, drinking motives are factored into endogenous variables, and the ego-energy is factored into a moderating variable. These variables will contribute to establishing more effective mental health strategies as a discriminating new factor in mental health.

A more comprehensive understanding of related factors influencing depression in an adult male population is needed. The researchers recommend future researchers to consider drinking motives, causes of stress, coping mechanisms, and relational factors of the population in a comprehensive manner.

## Data Availability Statement

The raw data supporting the conclusions of this article will be made available by the authors, without undue reservation.

## Ethics Statement

The studies involving human participants were reviewed and approved by Korea University Institutional Review Board (IRB). The patients/participants provided their written informed consent to participate in this study.

## Author Contributions

DY helped in conceptualization, methodology, software, data analysis, and writing the original draft preparation. HA helped in supervision, data analysis, writing, reviewing, and editing. Both authors contributed to the article and approved the submitted version.

## Conflict of Interest

The authors declare that the research was conducted in the absence of any commercial or financial relationships that could be construed as a potential conflict of interest.
